# Lipiodol marking for CT-guided radiofrequency ablation of adrenal aldosterone-producing adenomas: a case series

**DOI:** 10.1186/s42155-025-00614-2

**Published:** 2025-10-28

**Authors:** Hiromitsu Tannai, Sota Oguro, Hiroyuki Sakakibara, Yumi Nakajima, Hiroki Kamada, Yuta Tezuka, Yoshikiyo Ono, Hideki Ota, Kei Takase

**Affiliations:** 1 Department of Diagnostic Radiology, Tohoku University Hospital, 2‐1 Seiryo‐machi, Aoba‐ku, Sendai, Miyagi 980‐0874 Japan; 2 Diabetes, Metabolism and Endocrinology, Tohoku University Hospital, 1-1 Seiryo-Machi, Aoba-Ku, Sendai, Miyagi 980-8574 Japan

**Keywords:** Radiofrequency ablation, Primary aldosteronism, Adrenal venous sampling, Adrenal gland, Computed tomography

## Abstract

**Background:**

Tumor visualization during computed tomography (CT)-guided radiofrequency ablation (RFA) of adrenal adenomas is often limited by the small size of the lesion and needle-induced artifacts. These limitations can impair accurate needle placement and increase the risk of incomplete ablation. This report shows four adrenal RFA procedures in which iodized oil (Lipiodol®) marking enhanced lesion visibility.

**Case presentation:**

CT imaging and adrenal venous sampling showed unilateral right aldosterone-producing adenomas measuring 10–20 mm in patients with primary aldosteronism. Contrast enhancement of the nodules was confirmed via CT angiography after microcatheter insertion into the adrenal artery. Transarterial embolization was performed utilizing a 1:1 mixture of Lipiodol and iohexol. In three cases, Lipiodol deposition within the adenomas was confirmed on CT immediately post-embolization and persisted on the following day. Despite needle-induced artifacts during RFA, nodule visibility significantly improved. RFA was completed without major complications, which resulted in the resolution of primary aldosteronism and hypertension. In one case, although Lipiodol marking was discontinued due to the extravasation from the adrenal artery branch, RFA was completed on the following day.

**Conclusions:**

CT-guided RFA with Lipiodol marking facilitated the effective treatment of aldosterone-producing adrenal adenomas in three patients by significantly enhancing lesion visibility. Adrenal arterial embolization should be addressed with caution to avoid damaging the small blood vessels of the adrenal gland.

**Graphical Abstract:**

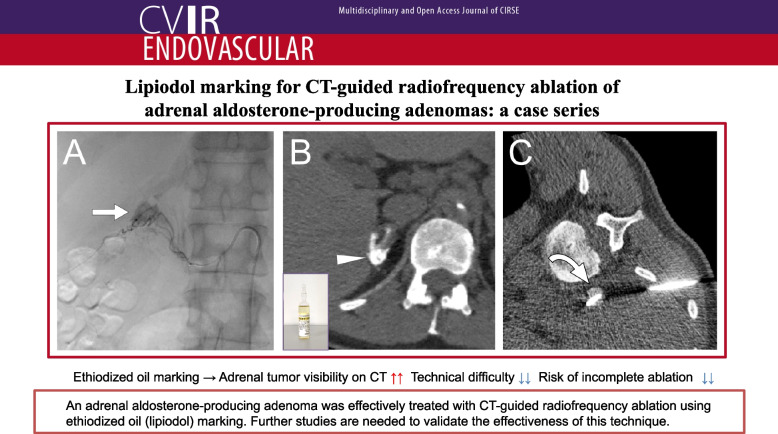

**Supplementary Information:**

The online version contains supplementary material available at 10.1186/s42155-025-00614-2.

## Background

Primary aldosteronism (PA) is a common cause of secondary hypertension, with aldosterone-producing adenomas (APAs) being a major underlying condition, typically managed through adrenalectomy [1, 2]. However, in recent years, less invasive computed tomography (CT)-guided radiofrequency ablation (RFA) has emerged as a treatment option [3]. Successful RFA relies on accurate needle placement within the lesion. Adrenal nodules often demonstrate poor visibility on CT, particularly for small lesions, with low-dose radiation protocols and in the presence of needle-induced metal artifacts [4]. This complicates needle guidance and increases the procedural difficulty of incomplete tumor ablation. Therefore, in this case report, we aimed to present four patients undergoing Lipiodol marking via transarterial embolization in order to enhance lesion visibility on CT prior to RFA. This technique, commonly employed in the treatment of renal cell carcinoma and hepatic cell carcinoma [5, 6], was adapted for use in these cases.

## Materials and methods

Patients were diagnosed with hypertension and PA according to guidelines [1, 2]. Segmental adrenal venous sampling was performed for PA subtyping. Patients with unilateral APA who were anatomically suitable for safe RFA were included according to the guidelines [1–5]. After obtaining informed consent, the patient underwent RFA preceded by transarterial Lipiodol marking.

Lipiodol marking was performed under angiography guidance a day prior to the RFA procedure. A 4-French vascular introducer sheath (Super Sheath; Medikit, Tokyo, Japan) was inserted into the right femoral artery, which was followed by selective cannulation of the suprarenal artery employing a modified shepherd hook catheter (Hanaco Medical, Saitama, Japan) and a 1.7–2.8-French tapered microcatheter (ASAHI Veloute; Asahi Intecc, Tokyo, Japan) using a 0.035 inch guidewire (Radifocus; Terumo, Tokyo, Japan) and a 0.016-inch micro guidewire (ASAHI Meister; Asahi Intecc, Tokyo, Japan). Digital subtraction angiography (DSA) was performed to confirm enhancement of the nodule. In some cases, subsequent CT angiography was performed utilizing an interventional radiology-CT system (Aquilion LB, Canon Medical Systems, Otawara, Japan). Embolization was performed utilizing a 1:1 mixture of iodized oil (Lipiodol, Guerbet Japan, Tokyo, Japan) and iohexol (Omnipaque 300, GE Healthcare Pharma, Tokyo, Japan), diluted to minimize imaging artifacts from undiluted Lipiodol. Non-feeding arteries were preserved whenever possible. After embolization, a plain CT scan was performed to confirm lipiodol deposition in the tumor. If the deposition was insufficient, embolization was repeated through another suprarenal artery.

The following day, CT-guided RFA was performed under deep sedation administered by an anesthesiologist as previously described [3]. Two 1.8 mm thick bipolar RFA applicators (CELON ProSurge applicator; Olympus, Tokyo, Japan) were inserted under intermittent CT fluoroscopic guidance. Tumor ablation was performed in some sessions while fine-tuning the needle position utilizing a multipolar RFA device (CELON POWER System, Olympus, Tokyo, Japan) with an output power of 40 W and delivering a total energy of 6 kJ [3]. After ablation, contrast-enhanced CT was conducted. Complete ablation was inferred from the absence of contrast enhancement in the surrounding adrenal parenchyma and adjacent liver tissue, although enhancement of the adenoma itself could not be definitively assessed caused by lipiodol deposition.

## Results

Four patients underwent adrenal RFA following lipiodol-marking. The patient’s characteristics before and after RFA are shown in Table 1. All patients were diagnosed with right unilateral APA, with a median diameter of 14.5 mm (range, 10–20 mm).

**Table 1:** Patient characteristics before and six months after RFA

Data	Case 1		Case 2		Case 3		Case 4	
Age	48		53		58		46	
Sex	F		M		F		F	
APA – size, side	10 mm, Rt		20 mm, Rt		14 mm, Rt		15 mm, Rt	
LI on AVS	35.7		70.6		92.9		33.9	
Complications related to lipiodol marking	—		—		—		Extravasation	
RFA technical success	Yes		Yes		Yes		Yes	
Complications related to RFA	—		—		Minor pneumothorax		—	
	Baseline	Post-RFA	Baseline	Post-RFA	Baseline	Post-RFA	Baseline	Post-RFA
Systolic BP, mmHg	150	103	140	124	125	90	119	120
Diastolic BP, mmHg	95	71	87	83	100	68	93	79
HR,/min	68	76	72	75	86	55	69	72
K, mmol/L	3.2	3.7	2.8	4.3	3.3	4.1	3.2	4.0
Number of HT drugs	1	1^a^	2	0	4	0	1	1^a^
K replacement, mmol/day	0	0	0	0	30	0	16.2	0
Creatinine, mg/dL	0.65	0.78	1.21	1.53	0.6	0.78	0.65	0.74
eGFR, mL/min/1.73m^2^	75	62	50	39	78	59	77	66
PAC, ng/dL	15.6	9.05	35.3	3.89	21.6	2.04	40.7	< 0.4^b^
PRA, ng/mL/h	0.2	2.0	0.2	2.3	0.3	0.6	0.5	0.8
ARC, pg/mL	1.12	10.69	1.8	9.75	2.46	3.53	2.07	1.59
ARR (PAC/PRA)	78	4.53	176.4	1.69	72	3.4	81.4	< 0.5^b^
ARR (PAC/ARC)	13.9	0.85	19.6	0.4	8.78	0.58	19.7	< 0.25^b^
Captopril ARR (PAC/PRA)	11.4	0.32	24.7	0.31	13.1	0.18	35	0.67
Saline infusion PAC, ng/dL	12.86	1.04	27.06	< 0.4^b^	—	—	19.6	< 0.4^b^
Biochemical outcome	Complete		Complete		Complete		Complete	
Clinical outcome	Partial		Complete		Complete		Partial	

LI is the aldosterone-to-cortisol ratio of the dominant side compared to the contralateral side. BP measurements were taken at the hospital. PAC was measured using the chemiluminescent enzyme immunoassay method. Outcomes are classified according to the primary aldosteronism surgical outcome (PASO) criteria

*APA* aldosterone-producing adenoma, *ARC* active renin concentration, *ARR* aldosterone to renin ratio, *AVS* adrenal venous sampling, *BP* blood pressure, *Egfr* estimated glomerular filtration rate, *HR* heart rate, *HT* hypertension, *LI* lateralization index, *PAC* plasma aldosterone concentration, *PRA* plasma renin activity, *RFA* radiofrequency ablation

^a^Denotes a reduction in dosage from the baseline

^b^Indicates the lower limit of quantification (LLOQ) or a value calculated using the LLOQ

**Fig. 1:**
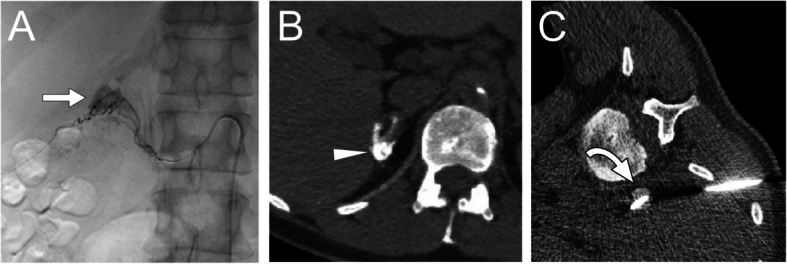
Lipiodol marking and RFA for right adrenal adenoma (Case 1). **A** Right adrenal arteriography from the microcatheter revealed contrast in the adrenal adenoma (arrow) and parenchyma. **B** Adrenal computed tomography (CT) image after lipiodol-marking. Lipiodol is deposited in almost of the adenoma (arrow) and part of the adrenal parenchyma. **C** CT fluoroscopy image during radiofrequency ablation. The high-density adenoma for lipiodol (arrow) is easy to visualize under the insertion of two thick needles. This image parameters were 120 kVp, 20 mAs, and three slices 2 mm thick

**Fig. 2:**
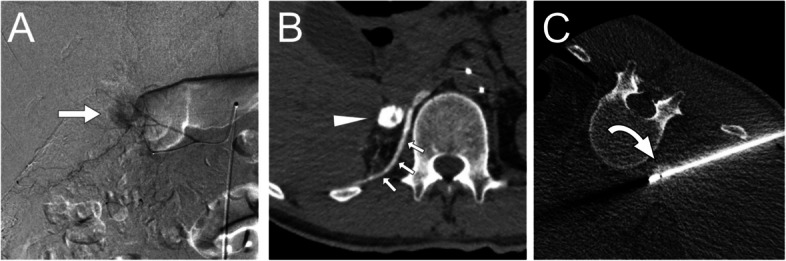
Lipiodol marking and RFA for right adrenal adenoma (Case 2). **A** Digital subtraction angiography image from a tumor-feeding branch of right middle adrenal artery shows the tumor stain of adrenocortical adenoma (arrow). **B** A computed tomography image after lipiodol-marking shows the lipiodol deposition in the adenoma (arrowhead) and diaphragm (small arrows). **C** CT fluoroscopy image shows a high-density adenoma (curved arrow) penetrated by an ablation needle. Lipiodol has been washed out from diaphragm

For lipiodol-marking, the middle suprarenal artery was embolized in Cases 1–3 (Figs. 1 and 2, Supplementary Fig. 1, and Table 2). On plain CT, lipiodol was sufficiently deposited in the adenoma and mildly in the adjacent adrenal parenchyma, perirenal adipose tissue and diaphragm. In Case 3, additional embolization of the superior suprarenal artery was performed. The median volume of lipiodol mixture used was 1.3 mL (range, 0.3–3 mL). No complications or symptoms occurred in these patients.

**Table 2:** Procedural details of Lipiodol marking for adrenocortical adenomas

Characteristics	Case 1	Case 2	Case 3	Case 4
Embolized side	Rt	Rt	Rt	Rt
Embolized suprarenal artery (origin)	M (Aorta)	M (Aorta)	M^a^ (Aorta) and S (IPA)	M (Renal artery)
Lipiodol mixture used, mL	0.3	3	1.3	NA
CT during angiography before embolization	+	+	–	–
Non-contrast CT after embolization	+	+	+	+
Technical success	Yes	Yes	Yes	No
Complication	None	None	None	Extravasation

*IPA* inferior phrenic artery, *S, M*, and *I* denote superior, middle and inferior suprarenal artery, respectively

*NA* not available

^a^Indicates non-tumor main feeder

The following day, lipiodol remained visible within the adenomas on CT imaging, while the lipiodol surrounding the adrenal gland had washed out from most regions. RFA was successfully performed without major complications in these cases. In Case 3, a slight pneumothorax developed during local anesthesia.

In Case 4, however, lipiodol was injected from the microcatheter to embolize the middle suprarenal artery. CT imaging revealed that most lipiodol and soft tissue density was concentrated along the diaphragm, indicating extravasation (Supplementary Fig. 3). Although the patient experienced no symptoms, the procedure was discontinued. The following day, extra adrenal hematoma and lipiodol decreased. RFA was successfully performed without any complications.

Six months after the procedure, PA was resolved in all patients. Hypertension was resolved in two patients and improved in two (Table 1). All patients remained under clinical follow-up.

## Discussions

In CT-guided RFA, repeated scanning necessitates a reduction in radiation dose per scan, which can compromise image clarity. The large gauge metal needle used for RFA creates artifacts on CT images, such as streaking and shading [6]. Adjusting the window width can reduce these artifacts and improve the visualization of the needle. However, this artifact and adjustment also diminishes the visibility of soft tissue, including small APAs with a mean diameter of < 20 mm [7, 8], which are typically well visualized on diagnostic CT. In some cases, small APAs can become nearly undetectable. In such cases, needle placement must rely on estimating the location of the lesion in relation to adjacent bone or soft tissue structures. However, adrenal nodules may be displaced rather than truly penetrated by the needle, and localization based solely on relative positioning can be inaccurate. This may result in incomplete ablation and persistent PA. Therefore, clear CT visualization of the APA itself is essential for procedural success.

Lipiodol marking is a well-established technique in CT-guided RFA or cryoablation for hepatocellular and renal cell carcinomas, which often exhibit poor visibility on non-contrast CT images [9, 10]. Previous studies show that Lipiodol deposition enhances local tumor control [10, 11]. Lipiodol marking is administered 1 or 2 days prior to the RFA procedure to prevent washout [10]. Regarding adrenal tumors, lipiodol deposition has been reported in typical hypervascular tumors, such as during chemoembolization for adrenal metastases from hepatocellular carcinoma and pre-RFA lipiodol marking for pheochromocytoma [12–14]. Hypervascularity of adrenocortical adenomas on CT angiography has also been described [15, 16], and in our cases, marked lipiodol accumulation was observed within the adenoma. This technique contributed to improved tumor visibility, accurate needle placement, and reduced procedural complexity.

Without Lipiodol marking, the accuracy of needle placement was occasionally suboptimal, and it was often necessary to compensate for misalignment caused by the initial bipolar needle insertion by adjusting the position of the second needle. Lipiodol marking enhances needle placement accuracy, thereby facilitating more precise targeting of the lesion. Therefore, adequate ablation of smaller tumors may be achievable with a single needle insertion. In contrast, in larger lesions, even if needle artefacts are present, the tumor is often visible relatively easily. Larger tumors retain a risk of incomplete ablation, omitting Lipiodol embolization might thus offer the advantage of confirming tumor enhancement presence or absence on post-ablation CT. Therefore, the decision to perform pre-RFA lipiodol marking for APA should be made considering the size of the tumor.

Furthermore, in patients with obesity, CT noise increases and image quality declines [17]; thus, this technique may be useful in such settings. Lipiodol marking might reduce tissue cooling effects and the risk of hemorrhage [11]. Therefore, this technique has the potential to improve clinical outcomes while reducing the incidence of complications during RFA.

The adrenal glands are supplied by numerous branches of the three main arteries: superior, middle, and inferior suprarenal arteries [18, 19]. These typically arise from the inferior phrenic artery, abdominal aorta, and renal artery, respectively. Anatomical variations have also been described, including a lack of one or more of these arteries or rare source of origins such as the celiac trunk, intercostal artery, gonadal artery, or accessory renal artery. Therefore, preprocedural CT mapping would be useful for the embolization.

In this review of a small number of cases, accurately identifying the tumor-feeding artery using DSA was challenging, and CT during adrenal arteriography was considered essential. Because the suprarenal arteries have branches, suppressing lipiodol distribution in the healthy adrenal parenchyma and selectively targeting the tumor may be possible in certain cases. However, considering the small diameter of the main suprarenal arteries (mean, 0.88–1.84 mm) [20], attempting selective embolization carries the risk of extravasation and is not always recommended, as in Case 4.

After RFA, at least 6 months of biochemical and clinical follow-up is required [21]. Imaging evaluation of the adenoma is not always mandatory; however, when assessing completeness of ablation, contrast-enhanced MRI may be useful, as lipiodol remains within the nodule after RFA and appears as a high-density on plain CT.

Other concerns have been raised regarding adrenal RFA employing Lipiodol marking. First, it is uncertain whether all adrenal adenomas adequately accumulate Lipiodol. Second, the effect on radiation exposure requires evaluation, as Lipiodol marking increases exposure, whereas RFA reduces it. These advantages and limitations should be carefully studied to determine their influence on treatment outcomes.

In conclusion, adrenal APAs could be effectively treated with CT-guided RFA using Lipiodol marking. Further studies are needed to validate the effectiveness of this technique.

## Supplementary Information

Supplementary Material 1.

## Data Availability

The datasets used and/or analysed during the current study are available from the corresponding author on reasonable request.

## Abbreviations

APAs: Aldosterone-producing adenomas
CT: Computed tomography
DSA: Digital subtraction angiography
PA: Primary aldosteronism
RFA: Radiofrequency ablation
