# Supplementary material for: Lipiodol marking for CT-guided radiofrequency ablation of adrenal aldosterone-producing adenomas: a case series
Source: CVIR Endovasc. 2025 Oct 28;8:95. doi: 10.1186/s42155-025-00614-2 (PMC12569250; doi:10.1186/s42155-025-00614-2)
Supplement: Supplementary file 1 — Supplementary Material 1. [file 42155_2025_614_MOESM1_ESM.docx]

**Supplementary Figure 1**

**Lipiodol marking and RFA for right adrenal adenoma (Case 3)**

**
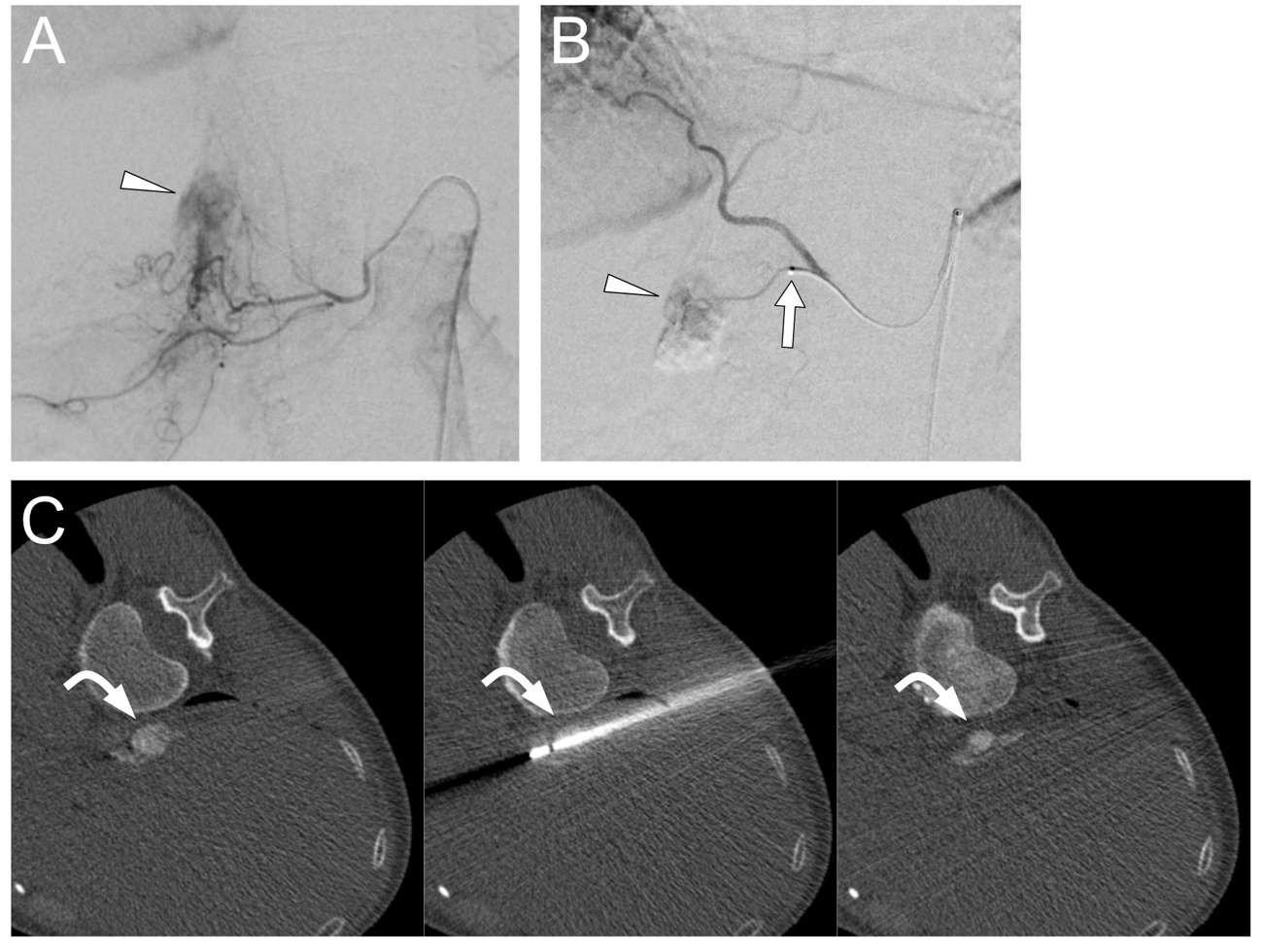
**

(A and B) Digital subtraction angiography (DSA) images from the middle suprarenal artery show the stain of adrenocortical adenoma (arrowhead). CT images after embolization indicate minimal Lipiodol deposition in the tumor. DSA from the superior suprarenal artery (arrow), branching from the inferior phrenic artery, demonstrates tumor stain. Lipiodol was also injected into this artery. (C) CT fluoroscopy images show high-density adenomas (curved arrow) penetrated by an RFA needle.

**Supplementary Figure 2**

**Contrast-enhanced CT after RFA (Cases 1–3)**


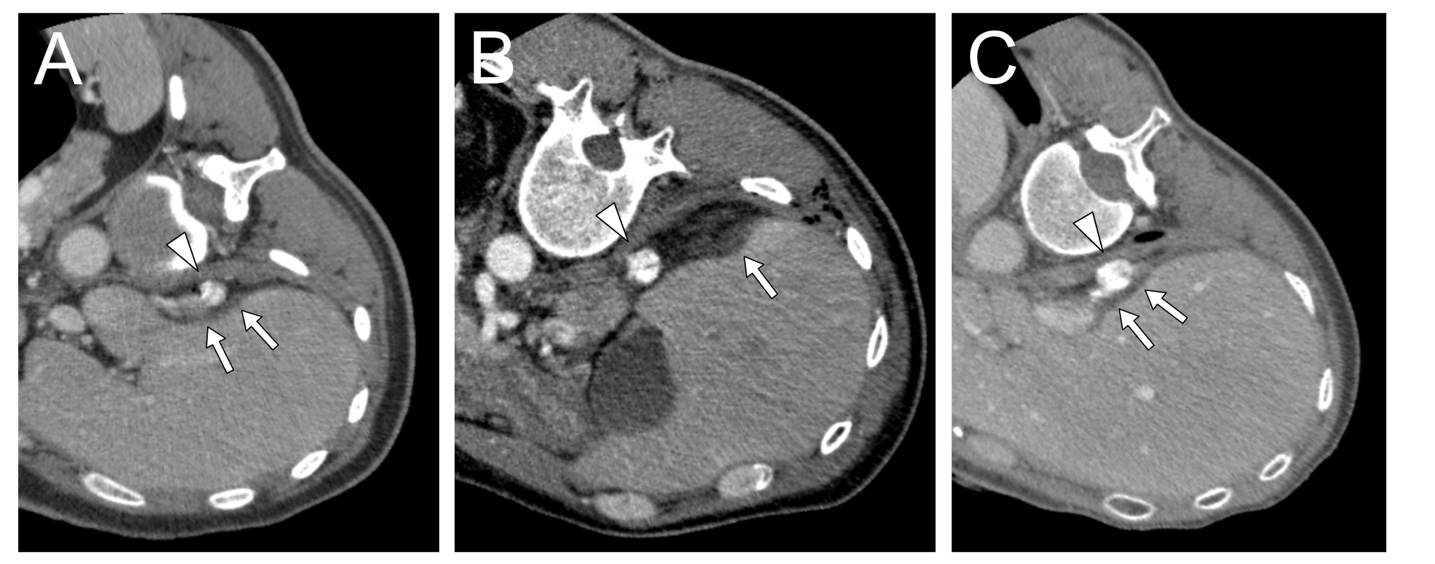


(A–C) Due to Lipiodol deposition, it is difficult to determine whether the adrenocortical adenomas (arrowheads) are contrast-enhanced. The unenhanced adjacent adrenal gland and liver (arrows) serve as internal references for confirming complete tumor ablation.

**Supplementary Figure 3**

**Lipiodol marking and RFA for right adrenal adenoma (Case 4)**


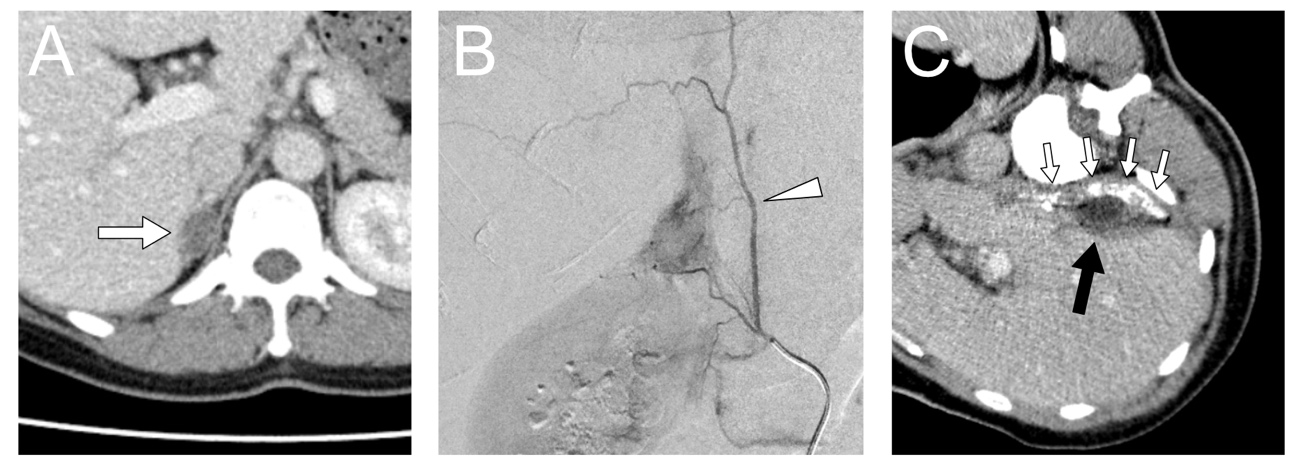


(A) CT image shows a low-density right adrenocortical adenoma (arrow). (B) Digital subtraction angiography image depicts fine branches of the middle and inferior suprarenal arteries (arrowhead). (C) Contrast-enhanced CT image obtained after RFA shows an unenhanced adenoma and adjacent liver parenchyma with extra-adrenal hemorrhage and Lipiodol deposition (small arrows) caused by lipiodol marking performed the previous day.
